# Balancing privacy and performance: the impact of facial defacing on AI in medical imaging

**DOI:** 10.1016/j.ebiom.2026.106457

**Published:** 2026-08-29

**Authors:** Yuli Wang, Yuwei Dai, Haoyue Guan, Cheng-Yi Li, Maulik Vora, Danica Ujano, Ayon Nandi, Jing Wu, Paul Zhang, Justin M. Honce, Chengzhang Zhu, Haris I. Sair, Leonora Balaj, Zhicheng Jiao, Yaou Liu, Cheng Ting Lin, Ihab Kamel, Li Yang, Harrison Bai

**Affiliations:** a Department of Radiology, University of Colorado Anschutz Medical Campus, Aurora, CO, USA; b Department of Radiology and Radiological Science, Johns Hopkins University School of Medicine, Baltimore, MD, USA; c Department of Neurology, Second Xiangya Hospital of Central South University, Changsha, Hunan, China; d Department of Computer Science, Johns Hopkins University, Baltimore, MD, USA; e Department of Radiology, Second Xiangya Hospital of Central South University, Changsha, Hunan, China; f Department of Pathology and Laboratory Medicine, University of Pennsylvania, Philadelphia, PA, USA; g School of Humanities, Central South University, Changsha, China; h Department of Neurosurgery, Massachusetts General Hospital, Boston, MA, USA; i Department of Diagnostic Imaging, Brown University Health, Providence, RI, USA; j Department of Radiology, Beijing Tiantan Hospital, Capital Medical University, Beijing, China

**Keywords:** Facial anonymization, Defacing algorithms, Medical imaging, Brain segmentation, Vision-language models

## Abstract

**Background:**

Recent NIH Data Management and Sharing (DMS) policy updates and NIH controlled-access data security requirements have increased attention to facial anonymization and controlled-access handling of shared head imaging data. This is particularly relevant for datasets submitted to or hosted by the Cancer Imaging Archive (TCIA), where NCI Cancer Imaging Program/TCIA implementation practices address imaging data containing potentially reconstructable facial anatomy. While intended to protect patient privacy and strengthen public trust, defacing can distort craniofacial geometry and alter image statistics, potentially compromising the fidelity and reproducibility of artificial intelligence (AI) models trained on such data. Existing studies primarily validate visual anonymization quality, but few have quantified its downstream impact on deep learning-based medical imaging tasks. Understanding this privacy-utility trade-off is crucial for responsible data sharing and compliant AI development.

**Methods:**

We systematically evaluated three representative defacing algorithms, two invasive (QuickShear and Py-Deface) and one less destructive, facial replacement (mri_reface), across MRI and CT datasets from 600 subjects spanning three institutions. Model performance was assessed on three clinically relevant applications: (1) brain segmentation and Evans ratio biomarker quantification in normal pressure hydrocephalus (NPH) MRI using SLANT and FreeSurfer; (2) representative-slice selection and diagnostic reasoning for brain tumour MRI using vision-language models (VLMs); and (3) automated emergency head CT report generation using a fine-tuned Otter-based vision-language model. Each method’s impact was quantified using Dice similarity, correlation metrics, reasoning accuracy, and natural-language generation scores (BLEU, METEOR, ROUGE, CIDEr).

**Findings:**

Invasive algorithms caused significant degradation across all tasks. QuickShear reduced mean Dice scores by up to 9% and introduced 14–19% failure rates during quality control, while PyDeface induced smaller but measurable performance losses. mri_reface maintained 100% success without any failures and achieved segmentation, diagnostic, and report-generation accuracy within 3–5% of the original data. Evans ratio distributions remained statistically consistent between mri_reface and original images (p > 0.05), whereas invasive methods introduced broader variance. Across all VLM tasks, mri_reface preserved high correlation with radiologist-selected slices (r = 0.979) and stable report-generation quality (BLEU-4 = 0.11 ± 0.06 vs. 0.12 ± 0.07 for original).

**Interpretation:**

Facial anonymization introduces a measurable privacy-utility trade-off that must be explicitly considered in the design of AI-ready medical imaging datasets. Invasive defacing compromises geometric and statistical integrity, reducing downstream model accuracy even outside facial regions. Facial replacement anonymization methods, such as mri_reface, effectively reconcile patient privacy with reproducibility, offering a practical path to NIH-compliant open data. Future regulatory and institutional policies should integrate quantitative privacy-utility assessment and mandate transparent reporting of anonymization pipelines to ensure that shared imaging data remain both ethically safe and scientifically valid under emerging digital health frameworks.

**Funding:**

This work was partially supported by the 10.13039/100000968American Heart Association (Award No. 25IPA1454088), the 10.13039/100000002National Institutes of Health (Award No. 1R03CA286693-01A1 and Award No. 1R01CA291826-01A1), the 10.13039/100000005U.S. Department of Defense (Award No. HT94252510807), and the 10.13039/100000001National Science Foundation (Award No. 2545071).

**Research in context**

**Evidence before this study**

Prior studies on facial anonymization and defacing have primarily focused on re-identification risk, anonymization success, and the effects of defacing on structural MRI analyses such as morphometry, segmentation, and brain measurement reproducibility. Several comparative studies (from 2010 to now) have shown that defacing methods differ substantially in their effects on image geometry and downstream quantitative analysis. However, most prior evaluations have been limited to structural MRI, with relatively little investigation of CT and minimal evidence regarding how defacing affects modern multimodal AI pipelines, particularly vision-language model (VLM) reasoning and report generation tasks.

**Added value of this study**

This study extends the existing defacing literature by evaluating the privacy-utility trade-off across multiple datasets, modalities, and AI task types within a unified framework. Using 600 imaging studies spanning NPH MRI, brain tumour MRI, and ED head CT, we compared invasive and less destructive, facial replacement defacing methods not only for segmentation and biomarker quantification, but also for representative-slice selection, vision-language diagnostic reasoning, and CT report generation. Our results broaden prior observations from structural MRI studies by showing that method-dependent defacing effects also remain relevant in multimodal AI settings.

**Implications of all the available evidence**

Taken together, existing literature and our results indicate that facial anonymization is not a neutral preprocessing step for head MRI/CT datasets with potentially reconstructable facial anatomy, particularly when submitted to TCIA or similar controlled-access repositories, although TCIA-compliant workflows should not be interpreted as a universal requirement for all NIH-funded studies. Invasive voxel-removal methods meet privacy goals but introduce geometric and intensity perturbations that propagate through alignment, feature extraction, and high-level VLM reasoning, risking underestimation of model performance and poor reproducibility once models are deployed on differently defaced data. Geometry-aware, facial replacement (such as mri_reface) appears to offer the best current balance: they satisfy facial anonymization requirements while preserving cranial topology closely enough for segmentation, biomarker extraction (Evans ratio), slice selection, and report generation to remain reliable. For institutions, this implies that (i) For datasets submitted to TCIA or similar repositories, less destructive facial-replacement anonymization may be preferable when available and when validated for the intended downstream task; (ii) AI models intended for public release should be trained and validated on already anonymised data; and (iii) data-sharing statements should report the exact defacing tool and version used so that downstream users can reproduce or adjust their own pipelines under future digital health and open-science mandates.

## Introduction

Maximising data sharing is widely recognised as a cornerstone of progress in data-driven research and the advancement of artificial intelligence (AI) and deep learning tools.^1^ Global collaboration and open dissemination of research materials, imaging data, and analytical pipelines have accelerated the translation of numerous AI-based medical devices that have received authorisation from the U.S. Food and Drug Administration (FDA).^2^, ^3^, ^4^, ^5^ The release of the NIH Policy for Data Management and Sharing (DMS) in January 2023^6^ reaffirmed the agency’s commitment to integrating transparency, reproducibility, and accessibility into the scientific process.

**Fig. 1:**
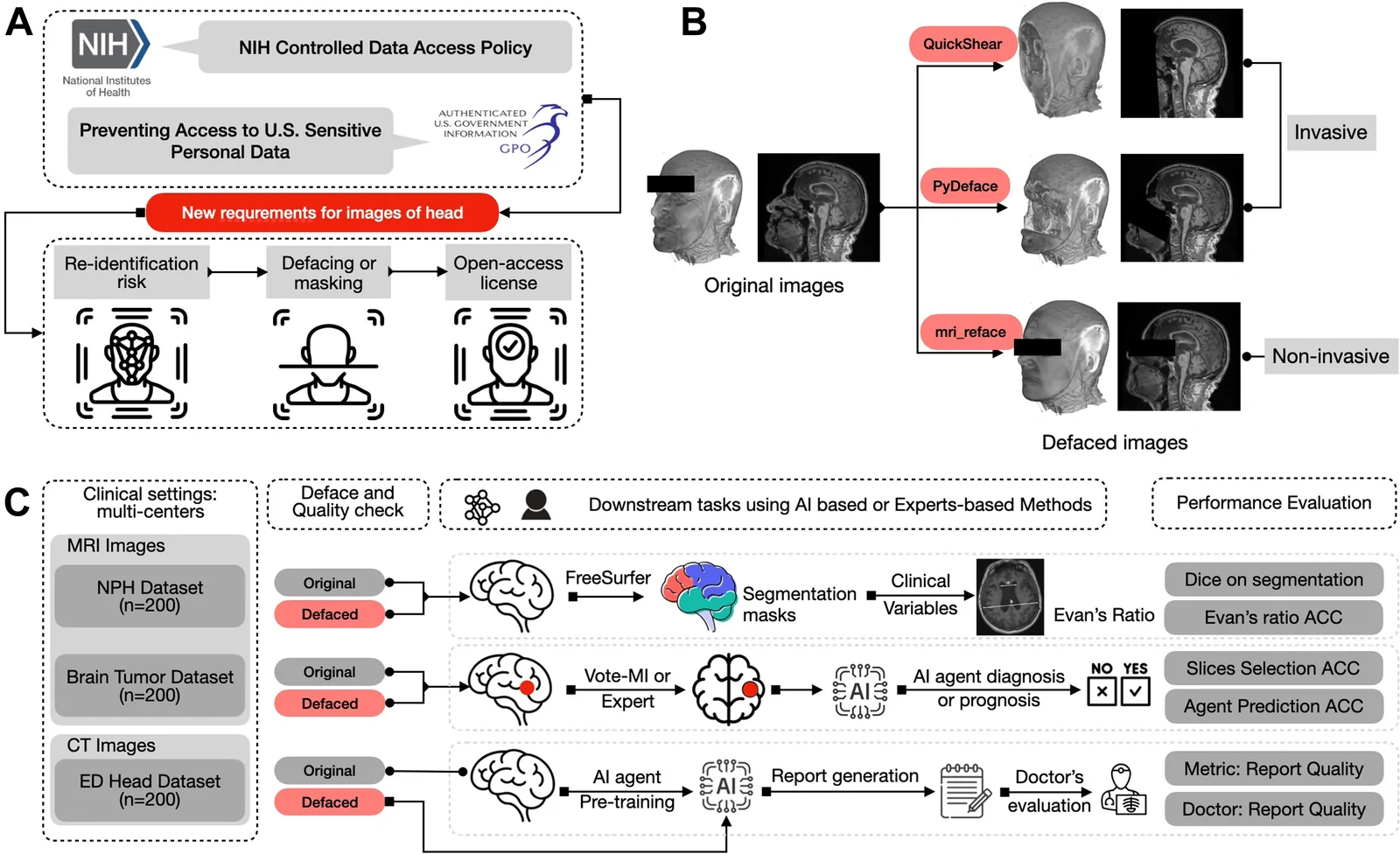
Assessment framework for evaluating the impact of facial defacing on AI model performance. (A) Recent NIH controlled-access data security updates and U.S. DOJ guidelines, together with NCI Cancer Imaging Program/TCIA implementation practices for datasets containing potentially reconstructable facial anatomy, have increased attention to privacy safeguards and controlled-access workflows for certain shared head imaging datasets. (B) Three representative defacing algorithms were evaluated: two invasive approaches that physically remove facial voxels (QuickShear and PyDeface) and one less destructive, facial replacement method (mri_reface) that replaces facial features. Corresponding rendered surface models and MRI examples are shown for each method. (C) Experimental framework for quantifying the effects of defacing across imaging modalities and downstream AI tasks. Three datasets were analysed: NPH MRIs, brain tumour MRIs, and head CTs from the emergency department (ED), each processed in both original and defaced forms. Structural analyses on the NPH dataset were performed using SLANT and FreeSurfer for segmentation and Evans ratio measurement. Diagnostic and prognostic reasoning on the brain tumour dataset employed Vote-MI or expert-annotated representative slices. Automated report generation from CT head scans was performed using a fine-tuned Otter-based vision-language model. Performance was comprehensively evaluated using Dice similarity, Evans ratio accuracy, slice selection correlation, diagnostic and prognostic accuracy, and report generation quality metrics, enabling systematic characterisation of defacing effects on downstream AI performance. For each cohort, the original images were processed independently by QuickShear, PyDeface, and mri_reface. The resulting image sets were then evaluated in parallel using the downstream task pipeline corresponding to that dataset.

Building upon this broader data-sharing framework, recent NIH controlled-access data security updates^7^^,^^8^ and U.S. Department of Justice guidelines^9^ have increased security expectations for certain controlled-access biomedical data repositories, while NCI Cancer Imaging Program/the Cancer Imaging Archive (TCIA) implementation practices specifically affect imaging datasets submitted to or hosted by TCIA that contain potentially reconstructable facial anatomy. As illustrated in Fig. 1 (A), these policy and repository-specific implementation changes reflect heightened privacy safeguards for shared head imaging datasets that could theoretically enable re-identification through advanced reconstruction or generative modelling techniques.^10^ In collaboration with dataset submitters, TCIA has implemented workflows, including defacing or controlled-access distribution, to support compliant sharing of affected imaging datasets.^11^

From a regulatory perspective, these measures substantially mitigate legal and ethical risks of re-identification^12^ and may strengthen public trust among institutions and patients,^13^ encouraging broader data deposition under controlled but transparent conditions. Yet, the broader scientific implications of defacing remain poorly understood. While essential for privacy protection, defacing may compromise AI model reproducibility and performance, effects that have not been systematically evaluated across diverse downstream applications such as segmentation, detection, diagnostic and prognostic modelling, and automated report generation.

Defacing typically removes or alters facial voxels to prevent re-identification, but in doing so can distort anatomical integrity, reduce interoperability across defacing methods, and erase critical DICOM metadata such as orientation or field-of-view information.^14^, ^15^, ^16^, ^17^ Prior studies have also shown that de-identification procedures can introduce measurable changes in structural brain measures and that some approaches may be either overly invasive or inconsistently effective across subjects.^16^^,^^18^ These alterations can impede image alignment and bias downstream feature extraction. As shown in Fig. 1B and C, we designed a reproducibility framework to systematically evaluate how facial removal affects AI pipelines.

Several prior studies have compared defacing methods in structural MRI and reported method-dependent effects on morphometric measurements, segmentation reproducibility, and processing robustness.^15^^,^^16^^,^^18^^,^^19^ More recent validation work in ADNI4 further showed that mri_reface was selected for implementation and had negligible effects on key brain measurements, supporting facial-replacement approaches for large-scale neuroimaging data sharing.^20^ At the same time, recent work has questioned whether defacing is a complete privacy solution in the era of diffusion-based reconstruction, while also suggesting that altered facial voxels may contain broader research value.^21^ However, most prior work has focused on structural MRI and conventional neuroimaging analyses, with limited evaluation across both MRI and CT and across modern downstream AI tasks such as vision-language model (VLM) reasoning and report generation. Although several defacing algorithms have been proposed,^10^^,^^22^, ^23^, ^24^ comparative evaluation across both MRI and CT and across modern downstream AI tasks remains limited. Here, we evaluate three representative methods: two invasive defacing approaches (QuickShear^25^ and PyDeface^26^), which anonymise images by removing or altering facial voxels, and a less destructive, facial replacement approach, mri_reface (^15^), which anonymises facial identity while attempting to preserve the original cranial and intracranial anatomy.

Our analysis includes MRI and CT data from 600 subjects across Johns Hopkins University and the University of Pennsylvania, encompassing three complementary applications: (i) regional brain segmentation and Evans ratio biomarker extraction in normal pressure hydrocephalus (NPH) MRI; (ii) representative-slice selection and diagnostic/prognostic reasoning in brain tumour MRI; and (iii) automated report generation from emergency department head CTs using fine-tuned VLM frameworks. Quantitative benchmarking and expert review were used to assess how de-facing influences segmentation accuracy, biomarker precision, diagnostic and prognostic validity, and report quality.

We found that (i) defacing algorithms exhibited variable failure rates across multiple modalities, (ii) defaced images caused measurable degradation across multiple AI tasks, including segmentation, biomarker quantification, disease classification, and report generation, and (iii) less destructive and facial replacement (mri_reface) methods maintained substantially higher fidelity, albeit with longer processing times. While major artifacts were identifiable through standard quality control, all approaches introduced subtle yet clinically meaningful reductions in model accuracy and interpretability.

Collectively, these findings demonstrate that defacing introduces a quantifiable privacy-utility trade-off that extends beyond simple visual alterations. Systematic evaluation of these effects is critical to ensuring that anonymization safeguards do not inadvertently compromise the reproducibility or clinical validity of medical AI systems, guiding both policy implementation and responsible data stewardship in the era of open, AI-driven medicine.

## Methods

### Datasets

This retrospective study was approved by the Institutional Review Boards of Johns Hopkins University (IRB00318113 for the NPH MRI dataset, IRB00452757 for the Brain Tumour MRI dataset, and IRB00424745 for the ED head CT dataset) with a waiver of informed consent. A data transfer agreement with the University of Pennsylvania (identification number: 73481-00) was also established for the Brain Tumour MRI dataset, under IRB protocol 851485. The study complied with the Health Insurance Portability and Accountability Act (HIPAA) and adhered to the Standards for Reporting Diagnostic Accuracy (STARD) guidelines. To avoid potential identifiability, facial renderings from unmodified images were removed or masked in all figures. All displayed visual materials were reviewed for privacy protection. Acquisition parameters were heterogeneous across institutions; available scanner and resolution details are summarised in Supplementary Table A1.

#### NPH MRI dataset

Imaging and clinical data were retrospectively collected from 803 patients diagnosed with normal pressure hydrocephalus (NPH) who underwent MRI and 534 patients with corresponding CT scans. MRI data were retrieved from the hospital’s Picture Archiving and Communication System (PACS; Carestream), and clinical metadata were obtained from the electronic medical record (Epic). For this study, a subset of 200 MRI scans was randomly selected to evaluate the impact of defacing algorithms on brain segmentation and biomarker quantification, focusing specifically on the Evans ratio.

#### Brain tumour MRI dataset

The BrainMD dataset and its associated benchmarks were developed as part of the study (Wang, Yuli, et al. (2024).^27^) The full BrainMD dataset is available under restricted access and can be obtained through a Data Use Agreement (DUA) with the Principal Investigator. It comprises 2453 annotated 3D brain MRI scans paired with radiology reports, medical records, and demographic information. All data were manually de-identified to remove protected health information (PHI) before downstream vision-language model experiments. For the present study, 200 subjects were randomly selected to assess the effects of defacing on model-based diagnostic and prognostic reading.

#### ED head CT dataset

We collected 33,128 head CT scans from 25,864 patients with ED who presented to Johns Hopkins Hospital and Johns Hopkins Bayview Medical Centre between May 1, 2017, and March 21, 2022. These scans were originally curated to pre-train the Otter-based vision-language model for automated CT report generation. For this study, a subset of 200 CT scans was randomly sampled to evaluate the influence of different defacing methods on model performance and report quality.

### Defacing algorithms

#### QuickShear

QuickShear removes facial features by defining a “shearing plane” that separates the brain from the face, setting all voxels anterior to this plane to zero. The plane is estimated from a brain mask by projecting the sagittal edge and fitting a convex hull to define the mid-sagittal separation line, which is then propagated across all sagittal slices. The spacing between the brain mask and the shearing plane can be adjusted to control the degree of facial removal. QuickShear was originally validated on the Kirby-21 scan-rescan data to assess defacing consistency. In our experiments, we used version 1.1.0 (March 23, 2017), with brain masks generated using BET with default settings.

#### PyDeface

PyDeface is a widely used Python neuroimaging tool for defacing because of its relative ease of installation and use. It uses a registration-based template-masking strategy. Specifically, PyDeface registers its template atlas to the input images and then applies the transformed facial mask to remove facial voxels. PyDeface does not perform morphological operations on the transformed facial mask. In this study, we used PyDeface version 2.0.0 with default settings.

#### mri_reface

mri_reface differs fundamentally from conventional defacing approaches by replacing, rather than removing, facial voxels. The average face is registered to each input image using ANTs, and the resulting deformation field is applied to preserve the subject’s brain while substituting only the facial region. Image intensities are harmonised through global and piecewise matching, followed by bias correction to achieve seamless blending. In preliminary method development, we examined mri_reface versions 0.2 and 0.3. All results reported in this study were generated using mri_reface version 0.3, which was selected for the final analysis because it produced more natural-appearing re-faced images.

### Brain segmentation methods

To assess the impact of defacing on automated brain segmentation, we applied two widely used whole-head MRI segmentation pipelines: SLANT^28^^,^^29^ and FreeSurfer.^30^ The overall workflow is illustrated in Appendix Fig. A.2. Briefly, NPH MR images served as inputs to both pipelines, each generating whole-brain segmentation masks. Ventricular structures, including the left, right, third, and fourth ventricles, were subsequently extracted for quantitative evaluation. Model-derived segmentation results were compared against ground-truth annotations manually delineated by a board-certified neurologist (initials: Y. D.) to compute Dice similarity coefficients and quantify defacing-related performance differences.

#### SLANT

The Spatially Localised Atlas Network Tiles (SLANT) framework segments brain MRIs using an ensemble of region-specific 3D CNNs, each trained on a spatially localised subvolume.^28^^,^^29^ Preprocessing includes affine registration, N4 bias correction, and intensity normalisation for spatial alignment. Outputs from all tiles are fused into a whole-brain map comprising 132 anatomical regions. SLANT (v1.0.3; https://github.com/MASILab/SLANTbrainSeg) achieves high intra- and inter-scan reproducibility across diverse MRI protocols.^28^

#### FreeSurfer

FreeSurfer^30^ provides automated cortical and subcortical segmentation from structural MRI. All analyses were performed using FreeSurfer version 7.3.2. We evaluated segmentation accuracy using the Dice similarity coefficient, a standard overlap metric for comparing two binary segmentation masks, defined as:

$$Dice=\frac{2\left|A\cap B\right|}{\left|A\right|\;+\;\left|B\right|}$$

A higher Dice score indicates better agreement between the automated segmentation and the expert reference, with a maximum value of 1.0 corresponding to perfect overlap.^31^

#### Evan’s ratio annotation

The Evans ratio, defined as the ratio of the maximal frontal horn width to the maximal internal skull diameter, was used as a quantitative biomarker for ventricular enlargement and diagnostic assessment of NPH.^32^ A board-certified neurologist (initials: Y. D.) with five years of experience in neuroimaging and NPH evaluation manually annotated Evans ratios for all original and defaced MR images.

For each case, the neuro-radiologist reviewed axial MR slices using a standardised DICOM viewer. The slice demonstrating the largest bilateral frontal horn diameter was identified, and the maximal width of the frontal horns was measured using a straight-line tool drawn between the most lateral margins of the frontal horns. The corresponding maximal internal skull diameter was then measured on the same slice at the level of the foramen of Monro. The Evans ratio was calculated as the ratio of these two measurements. All measurements were performed twice in separate sessions to assess intra-rater reliability, and any discrepancies greater than 5% were re-evaluated to ensure consistency.

### Slices selection methods

The brain Tumour MRI cohort was identified through random sampling, data cleaning, and inclusion criteria, yielding a final cohort of 2453 MRI studies from 561 distinct patients. From this cohort, 200 patients were randomly selected for analysis. Summary demographic characteristics of the selected cohort are shown in Appendix Table A.2.

#### Representative slice selection

We employed the Vote-MI algorithm,^27^ an efficient unsupervised method for representative 2D slice selection from 3D volumes. The method identifies diverse and representative slices in a single pass through the dataset, enabling efficient downstream use of 2D-based VLMs.

As detailed in our prior work,^27^ the Vote-MI pipeline consists of three major components: a) A patch-wise Variational Autoencoder (VAE)^33^^,^^34^ that performs unsupervised feature extraction; b) The Vote-MI algorithm, which identifies a diverse and representative subset of slices from unannotated data; and c) VLM-based reasoning tasks, which use the selected slices for diagnostic and prognostic tasks. We compared the performance of three VLMs-Flamingo,^35^ Med-Flamingo,^36^ and Med-PaLM-2^37^-under a zero-shot evaluation setting (shown as Appendix Fig. A.6), across three diagnostic tasks:

• “Presence of cancer in the image (yes/no)? (W/o cancer)”
• “Identification of brain cancer subtype (Cancer types)”
• “Describe the cancer status (improving, progressing, or no change)? (Cancer status)”

#### Task label distribution

We constructed three sets of task labels, including two diagnostic tasks and one prognostic task (“W/o cancer”, “Cancer types”, and “Cancer status”). The statistical distribution of task labels for the ground-truth of each sub-category is summarised in Appendix Table A.3. Each diagnostic and prognostic label was developed and validated by board-certified neurologists using MRI scans and corresponding radiology reports.

To qualitatively assess the visual impact of different defacing algorithms on facial anatomy preservation and information loss, we rendered 3D surface models from the Brain tumour MR images before and after defacing. We compared the visual appearance across four methods, QuickShear, PyDeface, mri_reface, and the Original (non-defaced) dataset, to evaluate how each algorithm modifies facial geometry. The results are shown in Appendix Fig. A.5.

### Vision language model for head CT report generation

#### Study design

We developed and fine-tuned a multimodal vision-language model based on the Otter architecture to automatically generate structured radiology reports from head CT images in ED settings.^38^ This framework was used to evaluate the impact of defacing on downstream report generation using three defaced datasets (QuickShear, PyDeface, mri_reface) and the original CT images.

#### Model architecture and training

The Otter framework integrates an MPT-7B language model^39^ with a CLIP ViT-L/14 vision encoder via a perceiver resampler and cross-attention mechanism. During fine-tuning, only the resampler and cross-attention layers were trainable, while all other parameters remained frozen to enhance efficiency and preserve generalisation. The model was trained for three epochs (approximately 12 h per model) using two NVIDIA A6000 GPUs. Training samples were formatted as image-instruction-report triplets, enabling multi-slice contextual reasoning over 3D (30-slice) inputs. The training workflow is illustrated in Appendix Fig. A.6.

#### Dataset preparation

For training, 33,128 3D head CT scans from 25,864 ED patients were sampled to ensure consistent 30-slice 3D inputs. For testing, 6000 slices from 200 CT scans (200 unique patients) were used. All test images were processed under the three defacing conditions (QuickShear, PyDeface, and mri_reface) as well as the original form.

#### Evaluation metrics

Model-generated reports were evaluated using standard natural-language generation metrics, including BLEU-1, BLEU-4, METEOR, ROUGE-L F1, and CIDEr-D, to assess lexical and semantic similarity to ground-truth reports. Comparative analyses across defacing conditions are shown in Appendix Fig. A.3E. Higher scores indicate greater lexical or semantic similarity between the generated report and the reference clinical report.

• BLEU-1^40^ measures unigram overlaps between the generated report and the reference report, reflecting word-level lexical similarity.
• BLEU-4^40^ measures overlap of up to 4-word sequences, capturing short-phrase consistency and local fluency relative to the reference report.
• METEOR^41^ evaluates alignment between generated and reference text using exact, stemmed, and synonym matches, providing a more flexible measure of semantic similarity.
• ROUGE-L F1^42^ measures the longest common subsequence between the generated and reference reports, reflecting how well the overall sentence structure and content ordering are preserved.
• CIDEr-D^43^ measures consensus-based similarity by weighting informative n-grams, emphasising clinically relevant phrases that are more distinctive in the reference report.

#### Physician evaluation

Because automated NLG metrics primarily measure text overlap and may not fully reflect clinical correctness or diagnostic completeness, we additionally performed blinded physician evaluation to assess report accuracy and interpretability. To assess the clinical quality and interpretability, three physicians (two radiologists (initials: M. V. and D. J.) and one neurologist (initials: Y. D.)) independently evaluated the machine-generated ED head CT reports. For each case, physicians were provided both the AI-generated report and the corresponding ground-truth clinical report retrieved from the radiology information system. Reviews were conducted in a blinded manner; evaluators were unaware of whether reports were derived from original or defaced images. Each report was rated on two dimensions: (1) clinical accuracy, defined as the correctness and completeness of key findings (e.g., haemorrhage, fracture, midline shift, mass effect); and (2) linguistic quality, reflecting grammatical coherence, clarity, and adherence to radiologic reporting conventions. Scores were assigned using a 6-point scale, where 1 indicated “unacceptable quality” and 6 indicated “clinically equivalent to ground-truth.” The detailed evaluation scores from each physician are summarised in Appendix Fig. A.7. Three physicians independently rated image quality under four conditions: QuickShear, PyDeface, mri_reface, and Original.

#### Intensity distribution

To quantitatively assess the impact of different defacing algorithms on voxel intensity distributions, we extracted intensity histograms from 3D CT volumes for each method: QuickShear, PyDeface, mri_reface, and the Original (non-defaced) dataset. Four examples of intensity distribution are shown in Appendix Fig. A.8. Each NIfTI image was loaded using NiBabel,^44^ and voxel intensities (in Hounsfield Units, HU) were extracted from the full 3D volume. The intensities were binned into histograms within the range of −1024 to +3000 HU to capture the full tissue dynamic range of head CT scans. Counts were normalised by the total number of voxels to enable inter-case comparison. The resulting histograms illustrate the distribution of soft-tissue and bone peaks under different defacing conditions. Red arrows in each panel mark the dominant soft-tissue peaks, serving as visual references for assessing potential global or local intensity shifts relative to the original images.

### Statistical analysis

All statistical analyses were performed using a paired design, since each original image was processed by all three defacing algorithms and the resulting outputs were evaluated on the same case. Quantitative comparisons between original and defaced conditions were performed using paired t-tests. Continuous variables are reported as mean ± standard deviation unless otherwise specified. Statistical significance was defined as p < 0.05, with significance levels denoted as ∗p < 0.05, ∗∗p < 0.01, and ∗∗∗p < 0.001. This retrospective study did not include a formal a priori power calculation; instead, the sample size was determined by predefined cohort selection from the available datasets, yielding a total of 600 images for comparative evaluation across tasks.

### Ethics

We would like to clarify that the requirement for informed consent was waived by the IRB due to the retrospective nature of the study, minimal risk to participants, and the use of previously collected clinical imaging data. Because this study specifically evaluates medical image defacing and anonymization methods, the investigators accessed original imaging data under IRB approval prior to application of de-identification procedures in order to assess residual facial identifiability risk and the impact of defacing on downstream imaging analyses and AI model performance. All data handling was conducted in accordance with institutional privacy and security requirements and HIPAA regulations.

### Role of funders

The funding bodies were not involved in study design, data collection, data analysis and interpretation, manuscript preparation, or the decision to submit for publication.

## Results

### Quality check

**Fig. 2:**
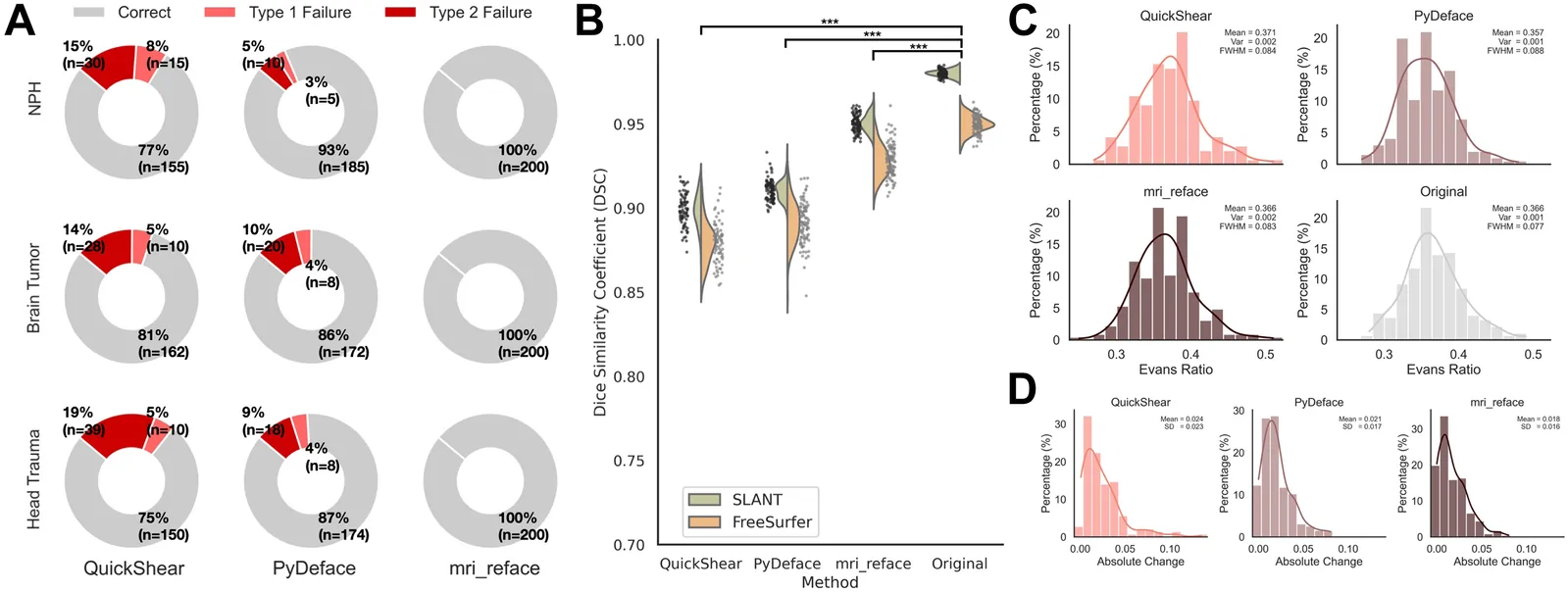
Quality control and diagnostic evaluation of defacing algorithms. (A) Visual quality assessment of three defacing algorithms: QuickShear, PyDeface, and mri_reface, applied to a total of 600 images (200 NPH MRIs, 200 brain tumour MRIs, and 200 emergency department head CTs). Each defaced image was manually reviewed through sequential inspection of axial slices from inferior to superior. Outputs were categorised as Success (facial features effectively removed), Type 1 Failure (facial structures remained), or Type 2 Failure (non-facial regions, such as brain tissue, inadvertently removed). (B) Ventricular segmentation performance using SLANT and FreeSurfer across original and three defaced image types (QuickShear, PyDeface, mri_reface). (C) Distribution of Evans ratios for NPH diagnosis across defacing methods. (D) Absolute percentage change in the Evans ratio relative to the original data. Statistical significance was denoted as ∗p < 0.05, ∗∗p < 0.01, and ∗∗∗p < 0.001.

As illustrated in Fig. 2 (A), we evaluated three defacing algorithms across a total of 600 imaging studies, comprising 200 NPH MRIs, 200 Brain Tumour MRIs, and 200 ED head CTs. Each defaced image underwent manual quality review through sequential inspection of axial slices from inferior to superior and back. Outcomes were categorised into three groups: Success, where facial identifying structures (e.g., nose, eyes/orbits, ears, mouth, and facial soft tissues) were adequately removed or replaced while preserving non-facial anatomy.; Type 1 Failure, where identifiable facial structures remained visible, either because the algorithm failed to detect the face or because facial removal was incomplete; and Type 2 Failure, where non-facial anatomy, including brain tissue, was inadvertently removed or altered. Among the evaluated methods, the invasive QuickShear algorithm exhibited the highest failure rates, with 14–19% Type 1 failures and 5–8% Type 2 failures across datasets. PyDeface exhibited moderate failure rates (5–10% Type 1 failures and 3–4% Type 2 failures), while the non-invasive mri_reface method achieved a 100% success rate, with no observed failures of either type.

### Results from NPH MRIs dataset

Fig. 2 (B) shows Dice score distributions for both methods across the original and three defaced image types (QuickShear, PyDeface, and mri_reface). On the original images, SLANT achieved a Dice score of 0.98 ± 0.002 and FreeSurfer 0.95 ± 0.005. Across defaced datasets, Dice scores were: QuickShear (SLANT 0.90 ± 0.010, FreeSurfer 0.88 ± 0.013), PyDeface (SLANT 0.91 ± 0.006, FreeSurfer 0.89 ± 0.013), and mri_reface (SLANT 0.95 ± 0.005, FreeSurfer 0.93 ± 0.010). All defacing methods produced statistically significant performance reductions compared with the original images (p < 0.05). Among them, mri_reface demonstrated the highest segmentation fidelity, with performance most closely matching the unmodified baseline. Representative segmentation masks are shown in Appendix Fig. A.3.

To evaluate the potential diagnostic implications, we further assessed how defacing affected Evans ratio measurements, a key biomarker for NPH, defined clinically as exceeding 0.3. A board-certified neuro-radiologist with five years of experience manually annotated Evans ratios for all original defaced MRIs. As shown in Fig. 2 (C), Quick-Shear (mean = 0.371, variance = 0.002, FWHM = 0.084, p < 0.05) and PyDeface (mean = 0.357, variance = 0.001, FWHM = 0.088, p < 0.05) introduced moderate distribution broadening relative to the original dataset (mean = 0.366, variance = 0.001, FWHM = 0.077), suggesting increased measurement variability with more invasive defacing. In contrast, mri_reface (mean = 0.366, variance = 0.002, FWHM = 0.083, p > 0.05) preserved the Evans ratio distribution, maintaining consistency with the original morphology. Fig. 2 (D) quantifies absolute deviations in Evans ratios relative to the original images. QuickShear produced the largest mean deviation (0.024 ± 0.023), followed by PyDeface (0.021 ± 0.017) and mri_reface (0.018 ± 0.016). The right-skewed error distributions indicate that while most cases exhibited minimal change (<0.03), a small subset of QuickShear-processed images showed substantial deviations.

### Results on brain tumour MRI dataset

For the brain tumour MRI dataset, representative slice selection was performed using the Vote-MI algorithm.^27^ The selected slices were subsequently analysed using three VLMs, Flamingo,^35^ Med-Flamingo,^36^ and Med-PaLM-2,^37^ for diagnostic and prognostic reasoning tasks. These tasks included (i) differentiating patients with cancer from those without cancer, (ii) identifying cancer subtypes among seven tumour categories, and (iii) classifying cancer status. Demographic characteristics of the patient cohort are summarised in Appendix Table A.2, and full experimental details are provided in the Methods Section. Statistical significance was assessed using paired t-tests for pairwise comparisons between the original and each defaced condition on the same cases. ∗ indicate significance levels as follows: ∗p < 0.05, ∗∗p < 0.01, ∗∗∗p < 0.001, and ns = not significant.

**Fig. 3:**
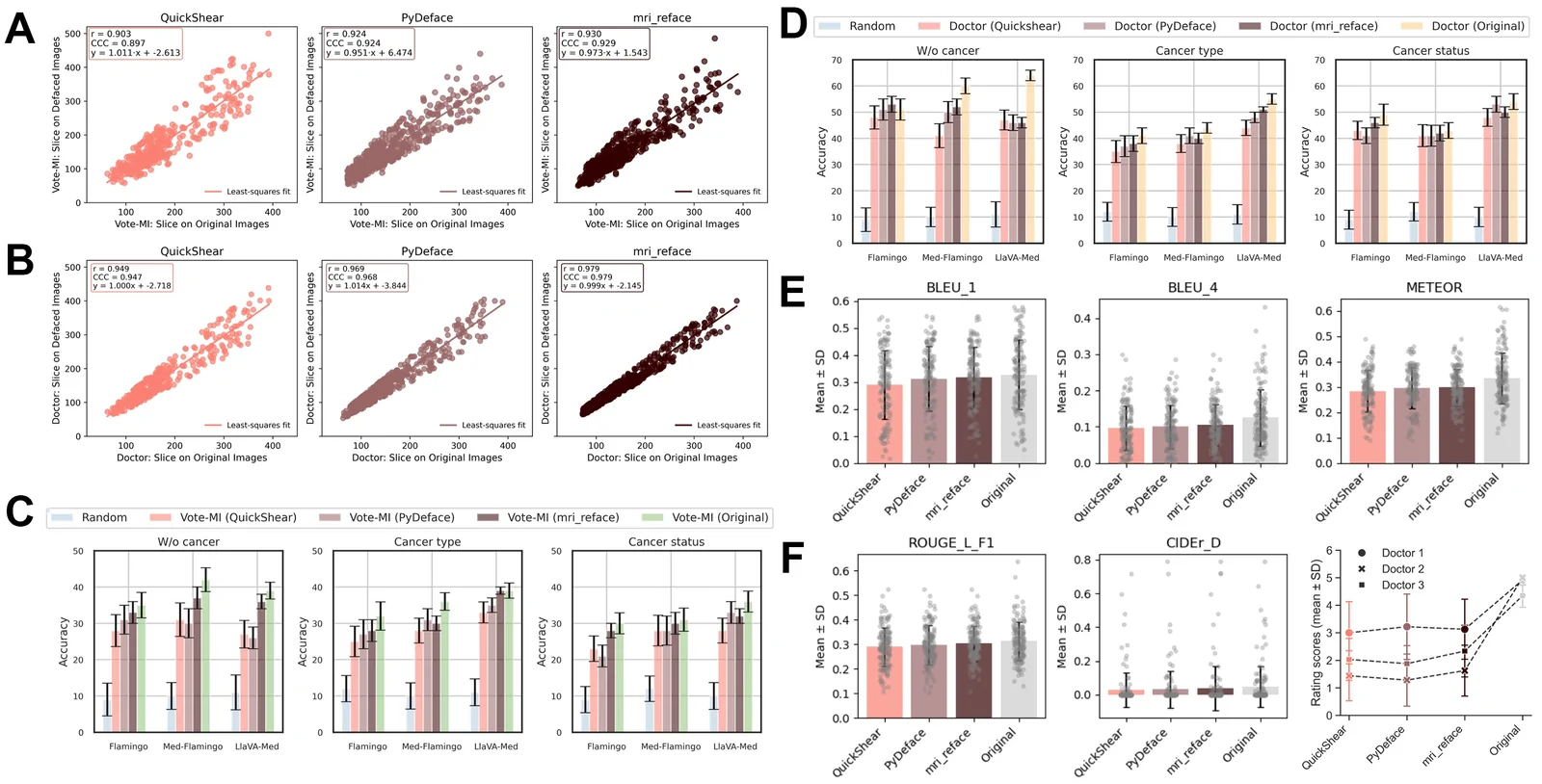
Results on the Brain Tumour MRIs and emergency department head CTs dataset. (A) Correlation between two independent Vote-MI slice selection runs on the same dataset. n = 200 per comparison group. (B) Comparison of slice selections made by two radiologists on corresponding images. Each point represents a single subject’s selected slice index, and a solid line denotes a least-squares regression fit. (C-D) Performance of three vision-language models (Flamingo, Med-Flamingo, and LLaVA-Med) on diagnostic reasoning tasks, including cancer presence, cancer sub-type, and cancer status classification. Slices were selected either by the Vote-MI algorithm (C) or by radiologists (D). Error bars represent standard deviation across the evaluated cases/tasks. (E) Performance of the Otter-based vision-language model for emergency department CT report generation across four image conditions: QuickShear, PyDeface, mri_reface, and original. Output quality was assessed using five standard natural language generation metrics (BLEU-1, BLEU-4, METEOR, ROUGE-L F1, and CIDEr-D). Additionally, three physician raters independently rated the clinical accuracy and linguistic fidelity of generated reports using a 6-point scale.

As shown in Fig. 3A and B, we evaluated the consistency of slice selection before and after defacing using Quick-Shear, PyDeface, and mri_reface algorithms. Panel (a) compares two independent Vote-MI slice selection runs on the original and defaced datasets, while panel (b) compares selections made by the same radiologist on the corresponding defaced images. Each point represents one subject’s selected slice index, with solid lines indicating least-squares regression fits. Strong correlations were observed across all methods, with mri_reface achieving the highest consistency (r = 0.930 for Vote-MI; r = 0.979 for radiologists), followed by PyDeface (r = 0.924 and r = 0.969) and QuickShear (r = 0.903 and r = 0.949).

Fig. 3C and D summarises the performance of the VLMs using slices selected by the Vote-MI algorithm and by radiologists, respectively. Each model was evaluated on three diagnostic reasoning tasks: (i) cancer presence, (ii) cancer subtype classification, and (iii) cancer status prediction. When Vote-MI selected slices were used (Fig. 3 (C)), the mean ± SD reasoning accuracy among three tasks for the original images was 38.8 ± 4.3%. Among the defaced datasets, mri_reface maintained the highest consistency (34.7 ± 4.9%), followed by PyDeface (29.1 ± 5.2%) and QuickShear (28.4 ± 6.1%), whereas random slice selection performed substantially worse (10.8 ± 2.6%). Fig. 3 (D) presents the corresponding results based on radiologist-selected slices. Overall accuracy improved across all conditions, with the original data reaching 55.9 ± 6.8%. mri_reface again preserved near-baseline performance (55.1 ± 7.1%), while PyDeface (52.8 ± 7.5%) and QuickShear (50.3 ± 8.2%) demonstrated moderate declines. Random selection remained markedly lower at 10.8 ± 2.6%.

### Results on ED head CT dataset

For head CT report generation, a key component of ED workflows, we fine-tuned the Otter model^45^ as a vision-language backbone to generate structured radiology reports from both original and defaced CT images. Detailed training configurations and experimental design are provided in the Method Section. Fig. 3 (D) summarises report generation performance across four image types (QuickShear, PyDeface, mri_reface, and original) using five standard natural language generation (NLG) metrics: BLEU-1, BLEU-4, METEOR, ROUGE-L F1, and CIDEr-D.

Across all metrics, mri_reface achieved results most comparable to the original images, with mean ± SD values of 0.31 ± 0.11 (BLEU-1), 0.11 ± 0.06 (BLEU-4), 0.30 ± 0.07 (METEOR), 0.30 ± 0.07 (ROUGE-L F1), and 0.04 ± 0.13 (CIDEr-D). PyDeface produced slightly lower scores (0.31 ± 0.12, 0.10 ± 0.06, 0.29 ± 0.08, 0.29 ± 0.08, and 0.03 ± 0.10, respectively), while QuickShear, the most invasive approach, yielded the lowest performance across all metrics (0.29 ± 0.13, 0.10 ± 0.06, 0.28 ± 0.08, 0.29 ± 0.08, and 0.03 ± 0.10). The original dataset achieved the highest overall scores of 0.33 ± 0.12, 0.12 ± 0.07, 0.33 ± 0.10, 0.31 ± 0.09, and 0.04 ± 0.12. To complement these quantitative findings, three physicians (two radiologists and one neurologist) independently evaluated the clinical accuracy and linguistic fidelity of the generated reports on a 6-point scale (Fig. 3 (E)). Ratings showed a consistent overall pattern, with reports generated from the original, non-defaced images achieving the highest scores across all three physicians (Doctor 1: 5.00 ± 0.00; Doctor 2: 4.91 ± 0.04; Doctor 3: 4.34 ± 0.40; overall mean: 4.75; p < 0.05). Among the defaced datasets, performance varied somewhat by reader. Doctor 1 scored PyDeface slightly higher than mri_reface, whereas the overall mean score across the three physicians was highest for mri_reface (2.35), followed by QuickShear (2.16) and PyDeface (2.13). The physician-specific scores were mri_reface: 3.10 ± 1.37, 1.63 ± 1.02, and 2.33 ± 0.98; PyDeface: 3.22 ± 1.57, 1.28 ± 1.02, and 1.88 ± 0.96; and QuickShear: 3.00 ± 1.48, 1.44 ± 0.97, and 2.03 ± 0.65 for Doctors 1, 2, and 3, respectively.

Across representative cases, QuickShear and PyDeface exhibited relatively larger variance in voxel intensity distributions, whereas mri_reface showed distributions closely aligned with those of the original images, as shown in Supplementary Fig. A8. In the physician evaluation, reports derived from the original, non-defaced images received the highest scores across all readers, indicating the best overall report quality. For the defaced datasets, the relative ranking varied somewhat by physician, although mri_reface achieved the highest mean score across readers. Detailed reader-specific scores are presented here to complement the physician evaluation criteria described in the Methods section and to provide a clearer link between the evaluation framework and the reported results.

## Discussion

Our findings collectively underscore an inherent and quantifiable trade-off between patient privacy protection and model performance in medical imaging AI. All tested defacing algorithms, whether invasive or non-invasive, introduced measurable degradation across deep learning models used for segmentation, diagnostic reasoning, and automated report generation. This decline stems from geometric and intensity distortions introduced during facial voxel removal or modification. Although these alterations are localised to non-diagnostic regions, they disrupt spatial continuity, image alignment, and statistical regularities that data-driven models rely on for generalisation. These results highlight a central challenge for the field: ensuring regulatory compliance and ethical data sharing while preserving the fidelity, interpretability, and reproducibility of AI systems trained on de-identified imaging data.

As privacy safeguards for shared head imaging data become more strict under evolving NIH/TICA controlled-access policies,^6^, ^7^, ^8^ understanding the downstream impact of facial anonymization becomes increasingly important. Without such evaluation, there is a risk that anonymised datasets may inadvertently bias or underperform in clinical applications, leading to reduced confidence in open data resources. Our results provide empirical evidence that even subtle modifications to craniofacial geometry can propagate through AI pipelines, affecting both low-level feature extraction and high-level reasoning tasks. These findings call for incorporating privacy-utility assessment into standard data curation and model validation frameworks, particularly for multi-institutional and regulatory-grade AI development.

Our findings are consistent with prior comparative defacing studies showing that defacing is not a neutral preprocessing step and that invasive methods can adversely affect downstream quantitative analyses. Earlier work in structural MRI reported method-dependent effects on morphometric measurements, segmentation reproducibility, and processing robustness, while also suggesting that facial-replacement approaches such as mri_reface better preserve downstream utility.^20^ Our results extend these observations beyond conventional structural MRI analyses^21^ by demonstrating similar privacy-utility trade-offs across segmentation, Evans ratio quantification, vision-language reasoning, and CT report generation.

The 100% success rate observed for mri_reface should be interpreted within the limits of our manual visual QC definition. In this study, success indicated that the visible face had been replaced and that no obvious original facial structures, such as the nose, orbits, ears, mouth, or facial soft tissues, were visually retained. However, manual review can detect only gross anonymization failures and cannot prove that all subject-specific facial information has been removed or that re-identification is impossible. Future studies should combine visual QC with quantitative privacy audits, including surface-distance analysis, face-recognition testing, and reconstruction-attack analyses, to more rigorously assess residual identifiability.

The three evaluated methods were selected to represent distinct anonymization strategies rather than to define the full performance envelope of all currently available tools. QuickShear was included as an aggressive removal-based method to probe a high-distortion end of the privacy-utility spectrum; PyDeface as a commonly used defacing baseline; and mri_reface as a facial-replacement approach. Accordingly, our conclusions should be interpreted as algorithm-specific and should not be generalised to all defacing algorithms. For intracranial-only applications, skull stripping may be reasonable when models are trained and evaluated entirely on skull-stripped data. However, it is not a universal substitute for facial anonymization because it removes all extracranial tissues, limiting research on calvarial, scalp, orbital, sinonasal, and other extracranial structures. It may also introduce a distribution shift for models trained on intact images, with performance depending on the skull-stripping tool used.

Notably, our study identifies non-invasive, facial replacement methods such as mri_reface as a promising solution to balance privacy and performance. Across all evaluated tasks, ventricular segmentation, disease classification, and report generation, mri_reface maintained accuracy within the smallest deviation from the original datasets while achieving complete anonymization. By preserving facial topology rather than physically excising voxel regions, mri_reface better preserves the overall head shape, image field-of-view, and continuity of non-facial anatomy. This may reduce preprocessing-related distribution shifts for models that were trained on intact head images. This demonstrates that privacy preservation does not necessarily entail a loss of scientific utility, provided that the anonymization approach is designed with structural awareness.

Our findings should also be interpreted alongside Mitew et al.,^46^ who showed that the destructive defacing tool PyFaceWipe preserved downstream volumetric performance well in their dataset. This suggests that destructive defacing is not always associated with poor downstream utility and that the impact of anonymization is method-and task-dependent. Accordingly, our results should not be interpreted as proving that facial-replacement methods are universally superior. Rather, among the methods and tasks evaluated in our study, mri_reface provided the most favourable balance between anonymization success and downstream performance.

These insights have several practical implications. First, institutions implementing defacing pipelines should adopt non-invasive or facial replacement algorithms whenever feasible. Second, AI developers should train or fine-tune models directly on anonymised data to enhance robustness to defacing-induced artifacts. Third, data-sharing consortia and regulatory agencies should encourage standardised benchmarking of anonymization techniques to ensure transparency and interoperability across datasets. Fourth, the emerging generation of privacy-preserving generative models-such as diffusion-based face replacement or synthetic data generation-offers a path forward for creating open datasets that satisfy both ethical and technical standards. Another limitation is that our volumetric evaluation in the NPH MRI cohort was restricted to ventricular structures. Although this choice was motivated by the clinical relevance of ventricular enlargement to the NPH task and the robustness of ventricle-based biomarker assessment, it does not fully capture the potential effects of defacing on smaller or more peripheral brain regions. Structures near the cortical surface may be more vulnerable to subtle alignment errors, boundary perturbations, or geometric inconsistencies introduced by anonymization. Future work should therefore extend the analysis to whole-brain regional volumetry and cortical or subcortical structures to provide a more comprehensive assessment of how defacing influences quantitative neuroimaging pipelines.

In conclusion, our study provides a systematic, quantitative assessment of how facial anonymization influences medical imaging AI across diverse tasks and modalities. The findings emphasise the need for careful design and validation of defacing workflows to maintain the reproducibility and clinical reliability of AI-driven tools. As open science initiatives expand under new data-sharing mandates, harmonising privacy protection with model integrity will be essential to sustaining public trust and advancing equitable, reproducible medical AI research.

## Contributors

Y. W. conceived and designed the method. Y.W. and Y.D. wrote the manuscript and did the data processing, experiment, and analysis. A.N., J.W., J.M.H., C.Z., H.I.S., L.B., Z.J., Y.L., C.Y.L., I.K., L.Y., and H.B. contributed materials and clinical expertise. C.T.L., I.K., L.Y., and H.B. supervised the work. All authors contributed to the interpretation of the results and editing of the final manuscript and provided technical support. Partial authors had access to data: Y.W., Y.D., M.V., D.U., H.B., and H.G. directly accessed and verified the internal data, and Y.W., P.Z., and H.B. verified the external data. All authors accept the final responsibility to submit for publication and take responsibility for the contents of the manuscript.

## Data sharing statement

The datasets used in this study are not publicly available due to institutional, regulatory, and patient privacy restrictions. Reasonable requests for data access may be directed to the corresponding author and will be considered subject to IRB approval and institutional data-use agreements. The custom code and scripts are available at the GitHub link available at https://github.com/YuliWanghust/deface_nih.

## Declaration of interests

We declare no competing interests.
