# Supplementary material for: Balancing privacy and performance: the impact of facial defacing on AI in medical imaging
Source: eBioMedicine. 2026 Aug 29;131:106457. doi: 10.1016/j.ebiom.2026.106457 (PMC13545401; doi:10.1016/j.ebiom.2026.106457)
Supplement: Supplementary file 1 [file mmc1.pdf]

Appendix A. Supplementary materials

Table A.1: Summary of imaging acquisition characteristics for the three datasets used in this study. JHH: Johns Hopkins Hospital

**Dataset**	**Institutions**	**Scanners**	**Modality**	**Sequence**	**Orientation**	**Voxel size / slice thickness**	**Cohort size**
NPH MRI dataset	JHH	Siemens	MRI	T1	Axial	0.5–5.0 mm	200
Brain tumour MRI dataset	JHH (BrainMD)	Siemens	MRI	T1	Axial	0.5–5.0 mm	200
ED head CT dataset	JHH	Siemens/GE	CT	–	Axial	1–5 mm	200

Appendix A.1. Defacing Failure Cases

Each defaced image underwent manual quality review through sequential inspection of axial slices from inferior to superior and back. Outputs were categorized into three quality classes: Success, where facial features were effectively removed; Type 1 Failure, where the algorithm failed to detect or process facial structures, leaving identifiable features intact; and Type 2 Failure, where non-facial regions, such as portions of brain tissue, were inadvertently removed. Representative examples of Type 2 failures are shown below.

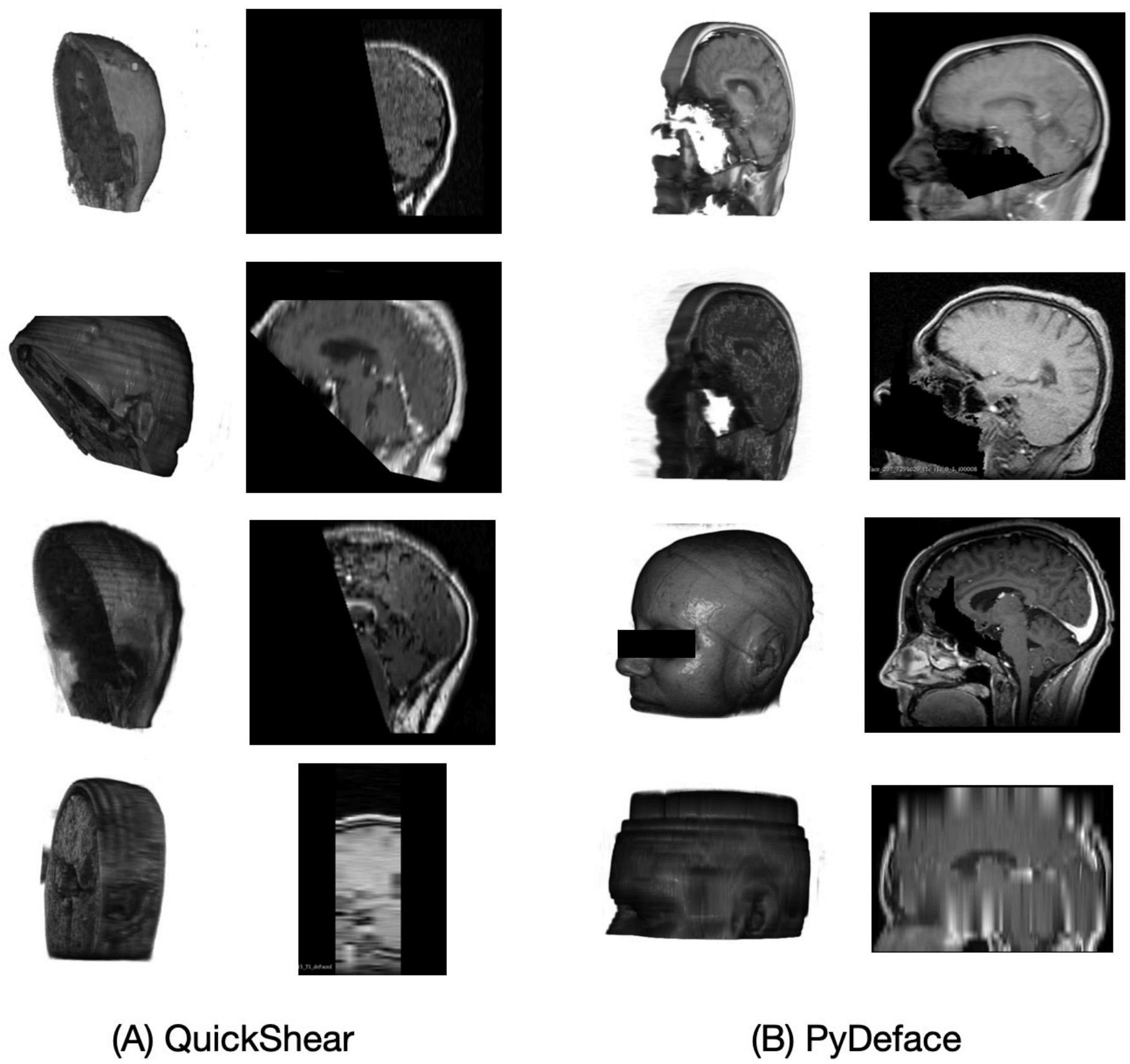

Figure A.1: Representative examples of Type 2 failure cases for (a) QuickShear and (b) PyDeface. In these examples, portions of non-facial brain tissue were inadvertently removed during defacing, demonstrating the excessive voxel removal characteristic of invasive anonymization methods.

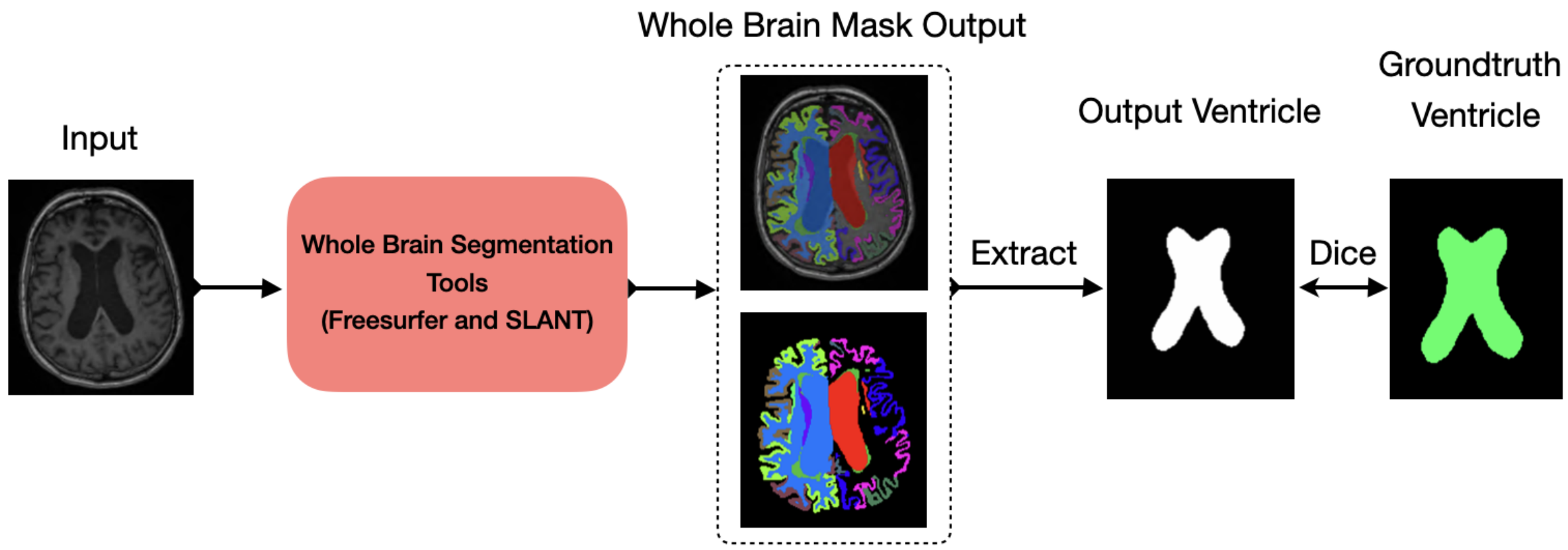

Figure A.2: Workflow of the brain segmentation pipeline used to evaluate the effects of defacing. Normal pressure hydrocephalus (NPH) MR images were processed using two established segmentation frameworks: SLANT and FreeSurfer. Each pipeline generates a whole-brain segmentation mask, from which ventricular structures (left, right, third, and fourth ventricles) were extracted for quantitative analysis. Ground-truth ventricular annotations were manually delineated by a board-certified neurologist, and segmentation performance was quantified using Dice similarity coefficients between model-derived and expert reference masks.

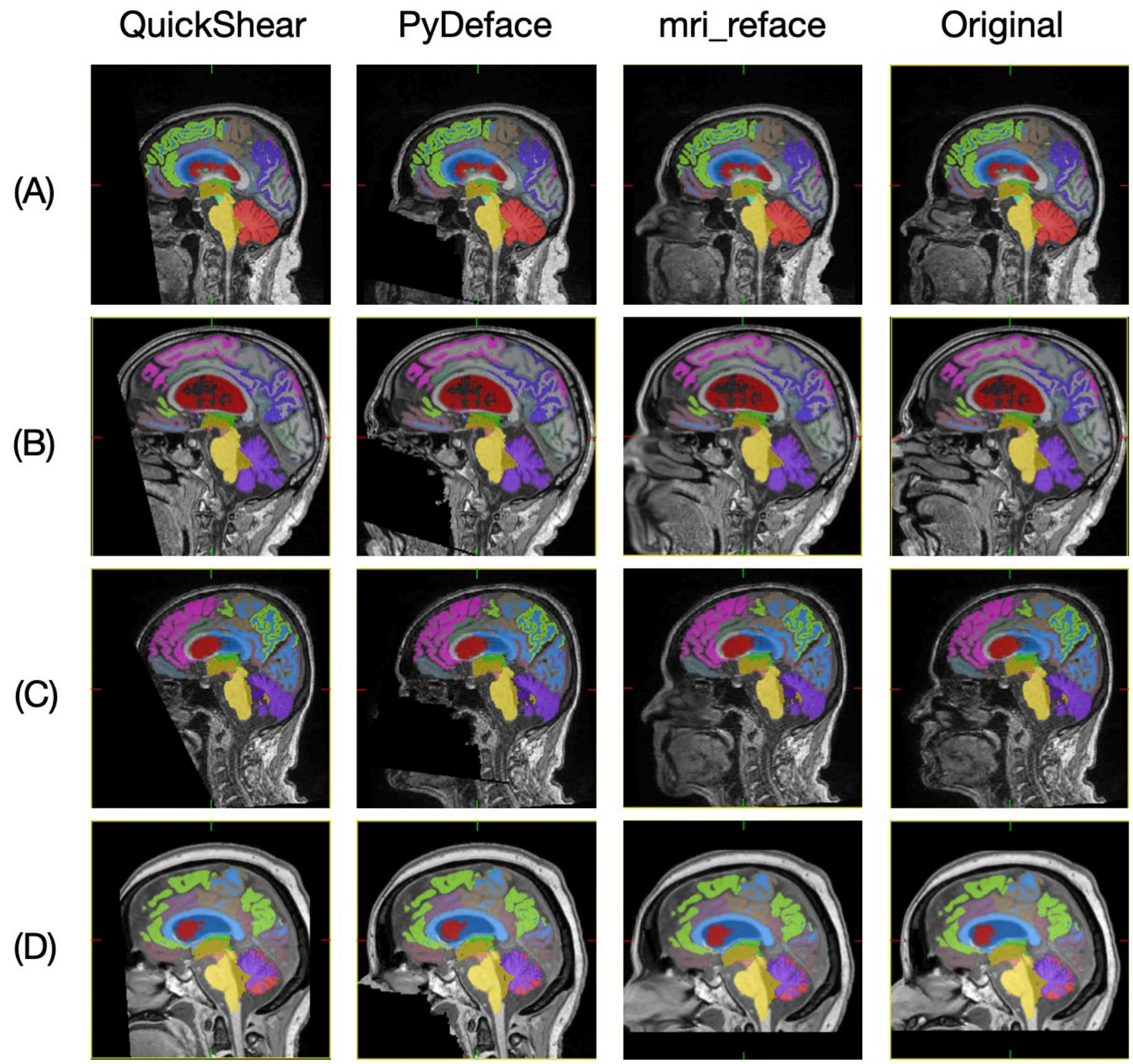

Figure A.3: Representative sagittal slices illustrating FreeSurfer segmentation results for the original, QuickShear-, PyDeface-, and mri_reface-processed MR images. Different brain regions are color-coded according to the FreeSurfer segmentation. Masks were spatially aligned and overlaid on corresponding MR images for visualization. Only ventricular regions were used for the quantitative segmentation accuracy analysis presented in the main text. (a) to (d) represents four different patients.

Appendix A.2. Demographic statistics

Table A.2: Demographic statistics of the proposed BrainMD dataset. Race and sex variables were derived from structured fields in the electronic health record. Race was analysed using EHR-recorded race categories; Sex was defined as sex recorded in the medical record.

**Demographics Statistics**		
**Attributes**		**All**
	Patients	200
**Sex**	Female	83 (43.0%)
	Male	84 (42.0%)
	Unknown	30 (15.0%)
**Age**	0-18	14 (7.0%)
	18-55	44 (22.0%)
	>55	138 (69.0%)
	Unknown	4 (2.0%)
**Race**	White	140 (70.0%)
	Asian	8 (4.0%)
	Black	10 (5.0%)
	Unknown	42 (21.0%)

Appendix A.3. Statistical distribution of diagnostic and prognostic labels

Table A.3: Statistical distribution of diagnostic and prognostic task labels in the BrainMD dataset (n=200 patients). LGG: Low-Grade Gliomas; GBM: Glioblastoma Multiforme; CPTs: Choroid plexus tumours

**Task Labels Statistics**			
**Types**	**Question**	**Answers**	**All**
			Patients 200
**Diagnosis**	**W/o cancer**	Yes	140 (70.0%)
		No	32 (16.0%)
		Uncertain	28 (14.0%)
**Diagnosis**	**Cancer type**	GBM	100 (50.0%)
		Glioma	58 (29.0%)
		LGG	20 (10%)
		Pineal tumours	6 (3.0%)
		Medulloblastoma	6 (3.0%)
		CPTs	4 (2.0%)
		Gangliocytoma	6 (3.0%)
**Prognosis**	**Cancer status**	Improving	18 (9.0%)
		Progressing	44 (22.0%)
		No change	138 (69.0%)

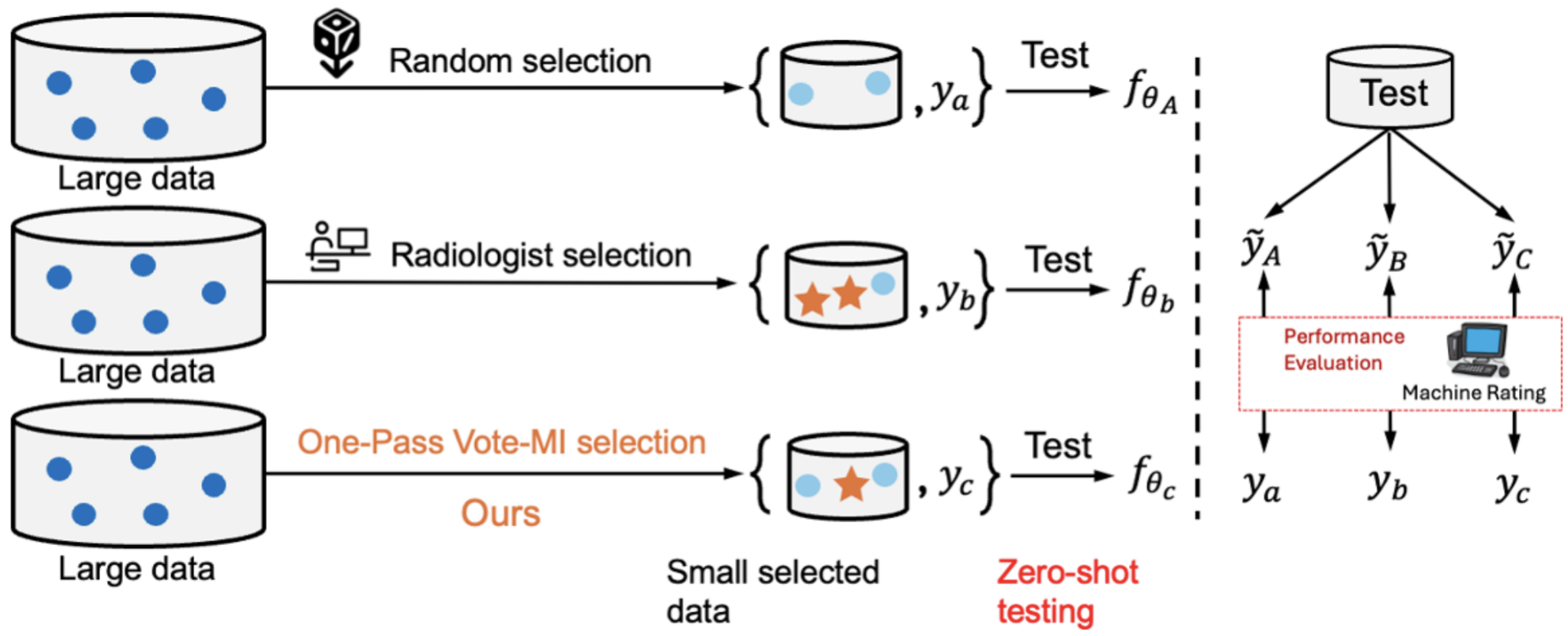

Figure A.4: Schematic framework of downstream zero-shot tasks. The one-pass Vote-MI representative 2D slice selection from 3D MRI volumes is compared with random and radiologist-selected slices.

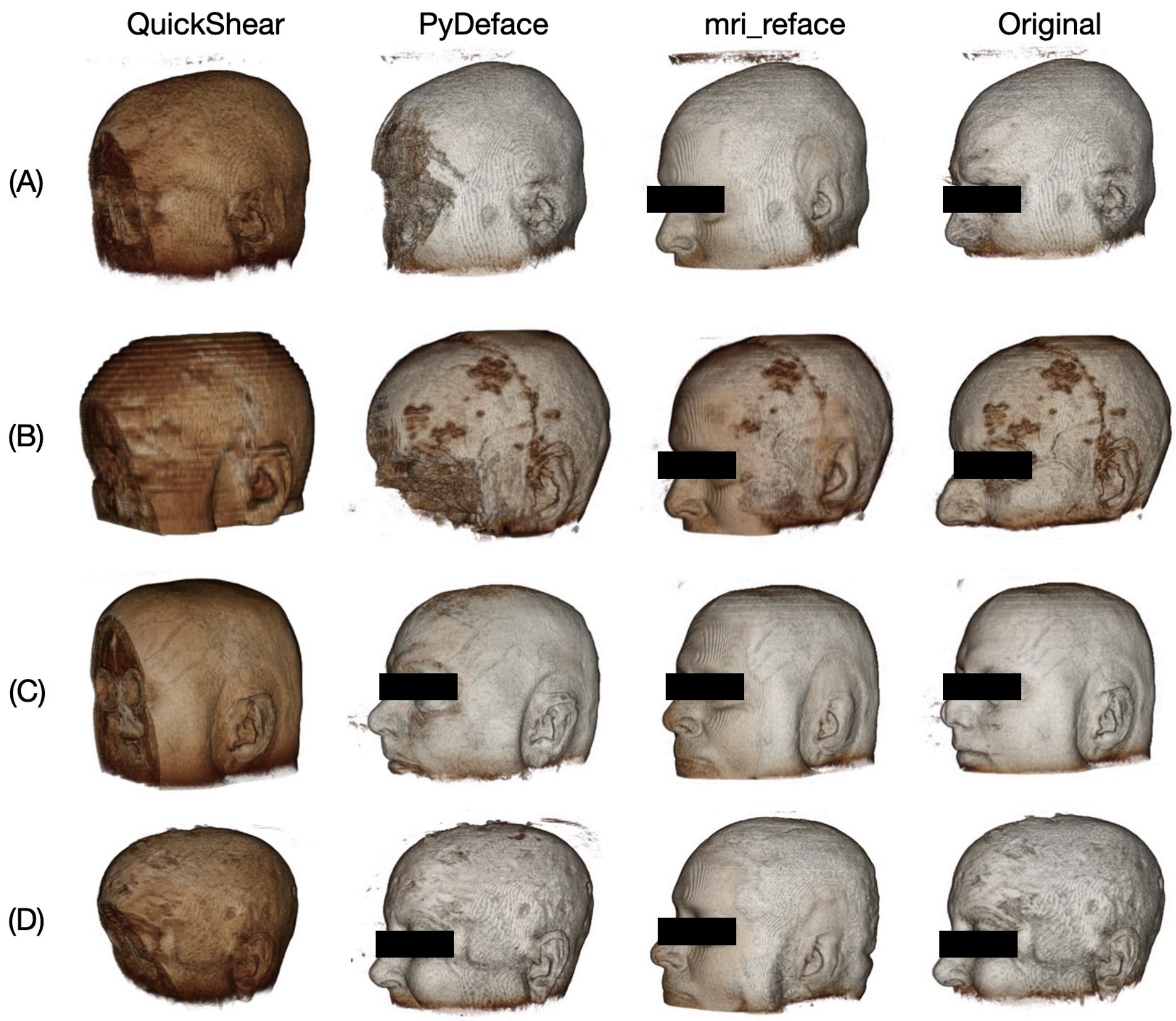

Figure A.5: Qualitative comparison of 3D-rendered head MR images after applying different defacing algorithms. (a-d) show four representative subjects processed using QuickShear, PyDeface, mri_reface, and the Original images.

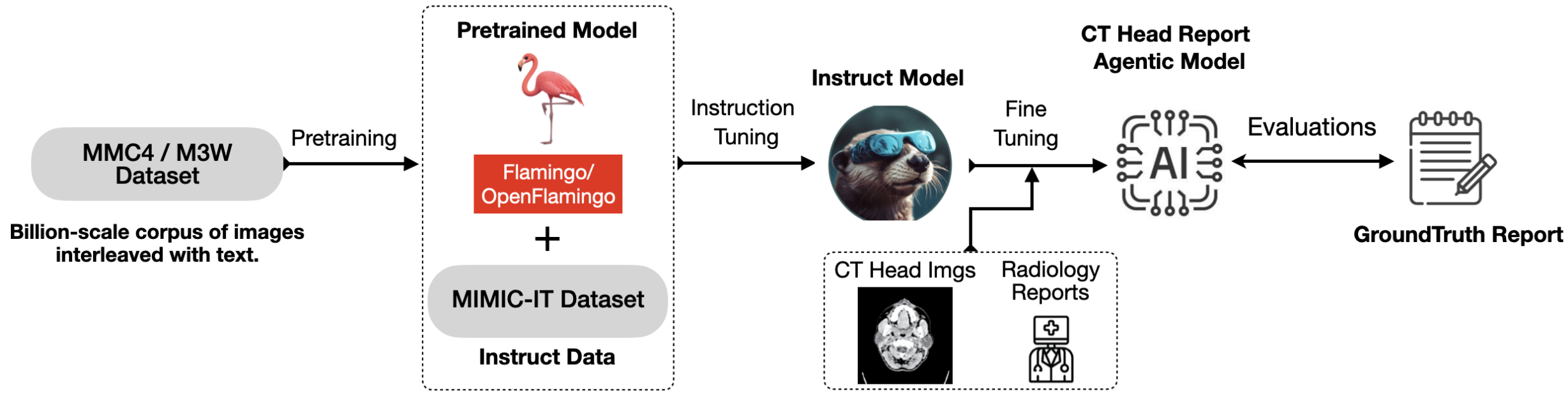

Figure A.6: Training pipeline for head CT report generation using multimodal, instruction-tuned large models. The process begins with large-scale multimodal pretraining on the MMC4/M3W corpus, a billion-scale dataset of image-text pairs, used to develop foundation models such as Flamingo or OpenFlamingo. The pretrained model is then instruction-tuned on the MIMIC-IT dataset, which aligns medical image-text reasoning through clinical instruction pairs. Finally, fine-tuning on the head CT trauma dataset enables domain adaptation for radiology report generation. The resulting Otter-based model produces automated CT reports that are quantitatively and qualitatively compared with ground-truth reports to evaluate accuracy, completeness, and clinical consistency.

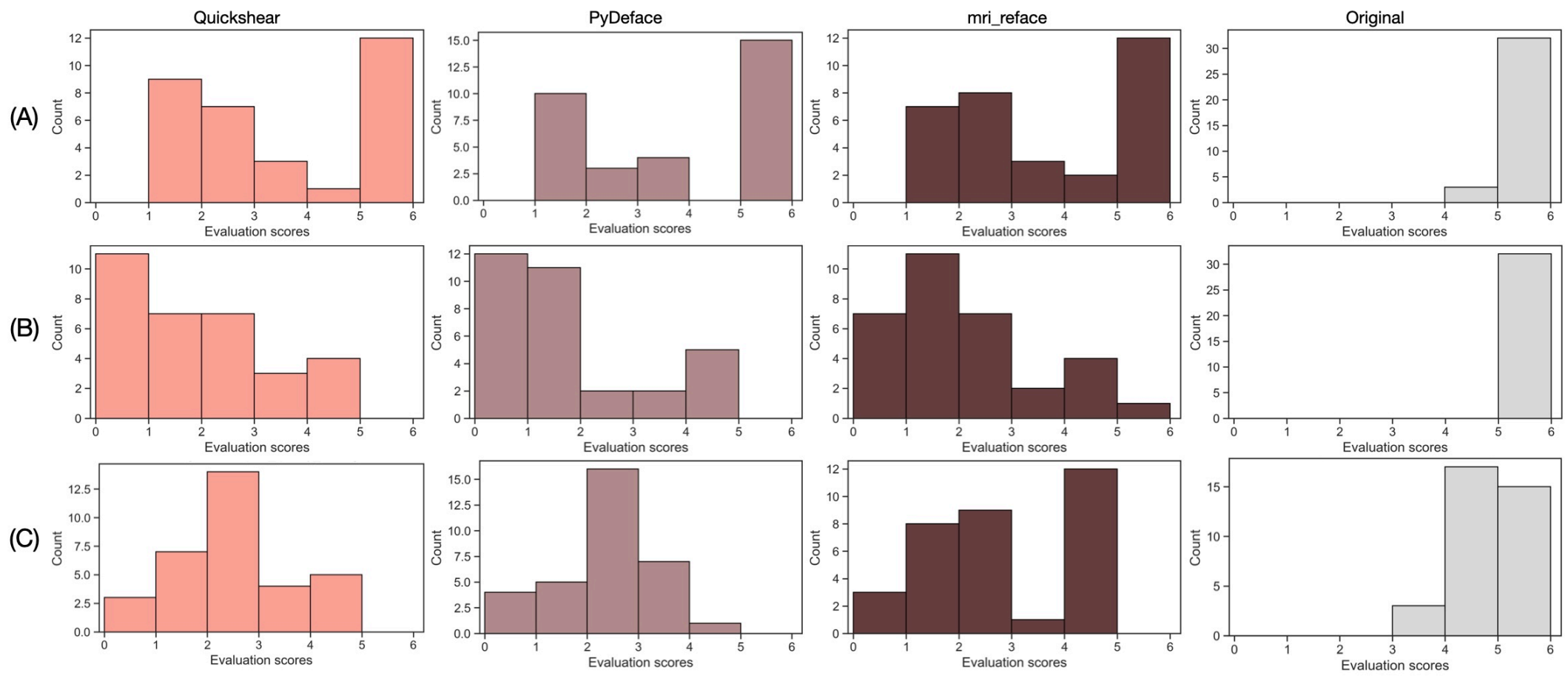

Figure A.7: Distribution of physician evaluation scores across defacing methods. Histograms depict the frequency of rating scores assigned by three physicians, (a) Doctor 1, (b) Doctor 2, and (c) Doctor 3, under four image conditions: QuickShear, PyDeface, mri_reface, and Original. Columns correspond to defacing methods, and rows represent each physician's individual scoring profile.

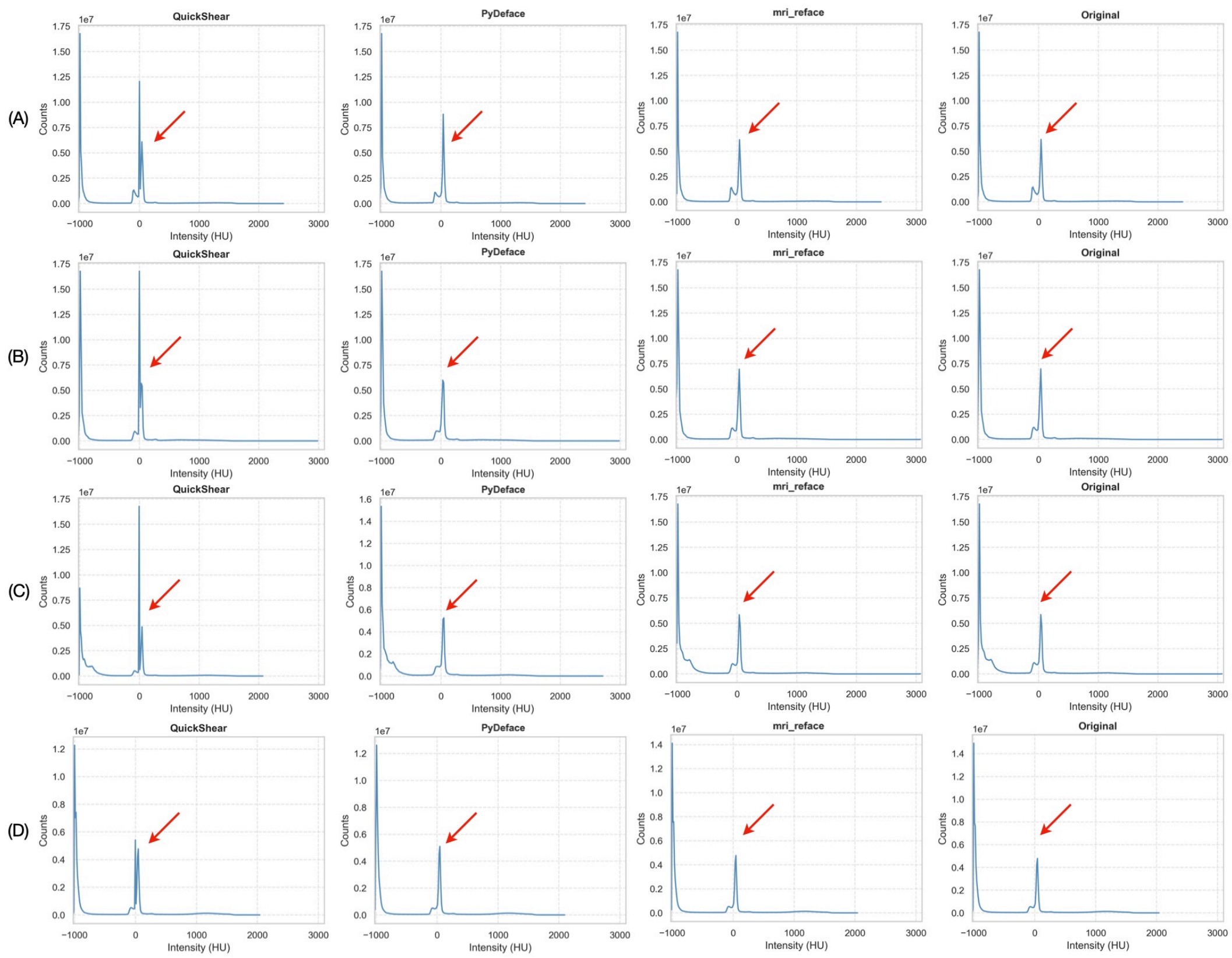

Figure A.8: Intensity distribution comparison of head CT volumes across four defacing methods. Each column represents four representative subjects, each showing histograms for QuickShear, PyDeface, mri_reface, and Original scans. The x-axis denotes voxel intensity (Hounsfield Units, HU) and the y-axis shows voxel counts. Red arrows mark the main soft-tissue peaks (around 0 to 100 HU). Consistent soft-tissue peaks across methods indicate preserved tissue contrast, whereas any shift or broadening of the peak suggests possible intensity perturbations introduced by the defacing algorithm.

Table A.4: Ventricular segmentation performance on the NPH MRI dataset. Dice similarity coefficient (DSC) values are reported as mean ± standard deviation. * indicate significance levels as follows: *$p < 0.05$, **$p < 0.01$, ***$p < 0.001$, and ns = not significant.

**Method**	**Segmenter**	**DSC (mean ± SD)**
QuickShear	SLANT	0.90 ± 0.010**
QuickShear	FreeSurfer	0.88 ± 0.013**
PyDeface	SLANT	0.91 ± 0.006*
PyDeface	FreeSurfer	0.89 ± 0.013*
mri_reface	SLANT	0.95 ± 0.005*
mri_reface	FreeSurfer	0.93 ± 0.010*
Original	SLANT	0.98 ± 0.002
Original	FreeSurfer	0.95 ± 0.005

Table A.5: Automated report-generation performance across original and anonymized head CT images. Values are reported as mean, variance, and standard deviation (SD).

**Dataset**	**Metric**	**Mean**	**SD**
PyDeface	BLEU_1	0.311915	0.119972
QuickShear	BLEU_1	0.290310	0.128401
mri_reface	BLEU_1	0.314836	0.112457
Original	BLEU_1	0.325007	0.129737
PyDeface	BLEU_4	0.100087	0.059785
QuickShear	BLEU_4	0.095979	0.060679
mri_reface	BLEU_4	0.113066	0.063361
Original	BLEU_4	0.124805	0.067616
PyDeface	METEOR	0.294175	0.080397
QuickShear	METEOR	0.283684	0.082484
mri_reface	METEOR	0.306524	0.074569
Original	METEOR	0.325142	0.097439
PyDeface	ROUGE_L F1	0.297411	0.078786
QuickShear	ROUGE_L F1	0.290383	0.078511
mri_reface	ROUGE_L F1	0.302918	0.071232
Original	ROUGE_L F1	0.311438	0.090617
PyDeface	CIDEr_D	0.032364	0.108360
QuickShear	CIDEr_D	0.029828	0.102118
mri_reface	CIDEr_D	0.039911	0.130985
Original	CIDEr_D	0.042581	0.122748

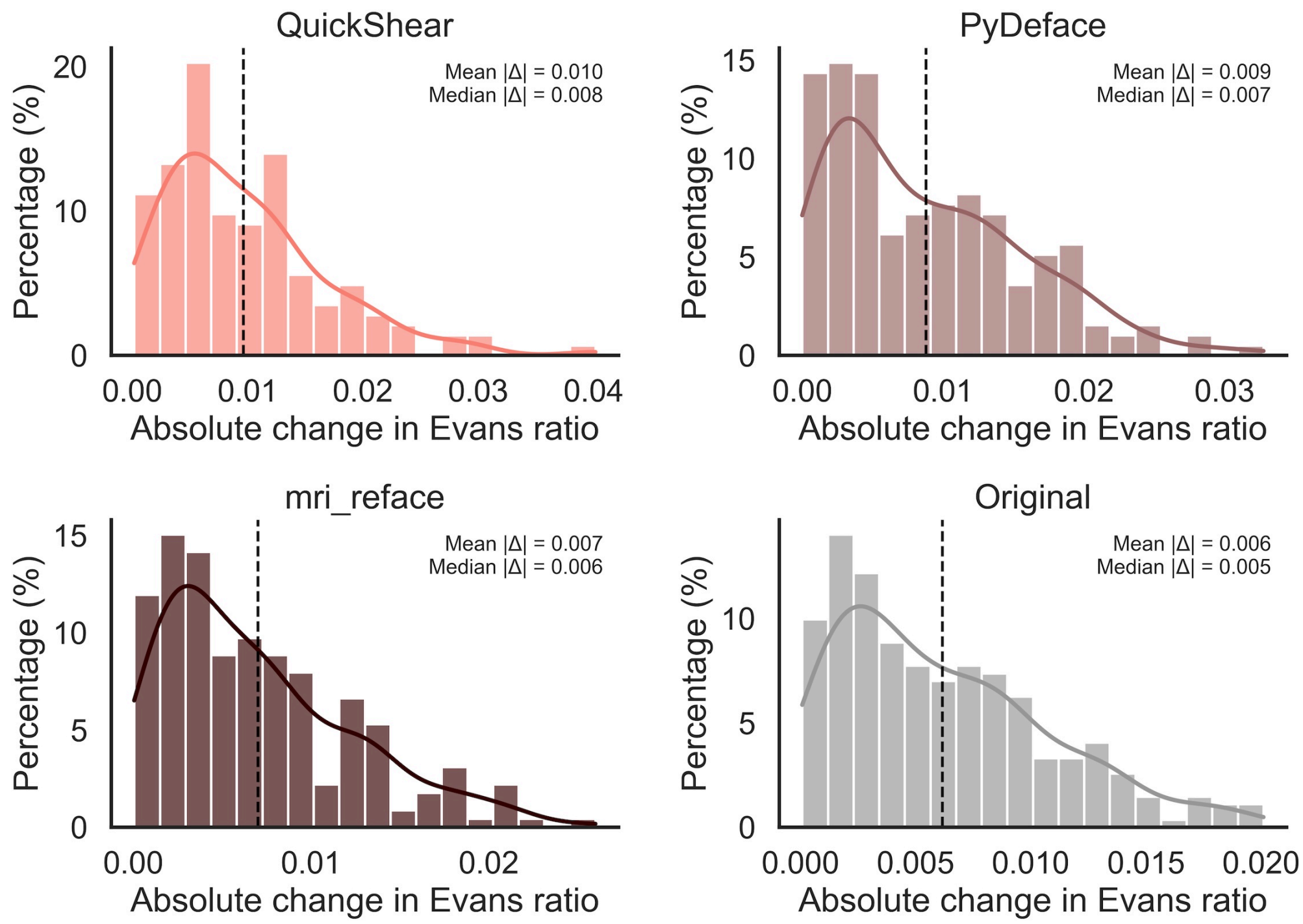

Figure A.9: Intra-reader repeat annotation: distribution of absolute change in Evans ratio across defacing conditions. Histograms show the distribution of the absolute difference between the first and second Evans ratio annotations for Quickshear, PyDeface, mri_reface, and original images. Dashed vertical lines indicate the mean and median absolute change in each group. Repeat-measurement differences were small overall, but the distributions for Quickshear and PyDeface were shifted slightly toward larger changes compared with mri_reface and the original images, indicating modestly greater intra-reader variability under the more invasive defacing conditions.

Appendix A.4. Repeat-measurement sensitivity analysis of manual Evans ratio annotation

To determine whether the observed Evans ratio differences could be explained by reader variability rather than by the defacing process itself, we performed an additional repeat-measurement sensitivity analysis. The Evans ratio was manually measured by radiologists using the standard definition: the maximum frontal horn width of the lateral ventricles divided by the maximum inner skull diameter on the same axial slice. Because manual measurement should be relatively robust unless defacing alters anatomical features relevant to the skull boundary, we generated a second annotation set by introducing small random perturbations around the original Evans ratio measurements to simulate repeat reads. We then assessed first-versus-second measurement agreement across Quickshear, PyDeface, mri_reface, and original images using three complementary analyses: absolute change distributions, paired scatter plots, and Bland–Altman plots. Overall, repeat-measurement variability remained low across all groups, with mean absolute changes of 0.010 for Quickshear, 0.009 for PyDeface, 0.007 for mri_reface, and 0.006 for the original images. Correlations between first and second measurements were high in all conditions, and Bland-Altman analysis showed near-zero mean bias without evidence of systematic directional shift. However, Quickshear and PyDeface showed slightly wider 95% limits of agreement than mri_reface and the original images, indicating modestly increased repeat-measurement variability under the more invasive defacing methods.

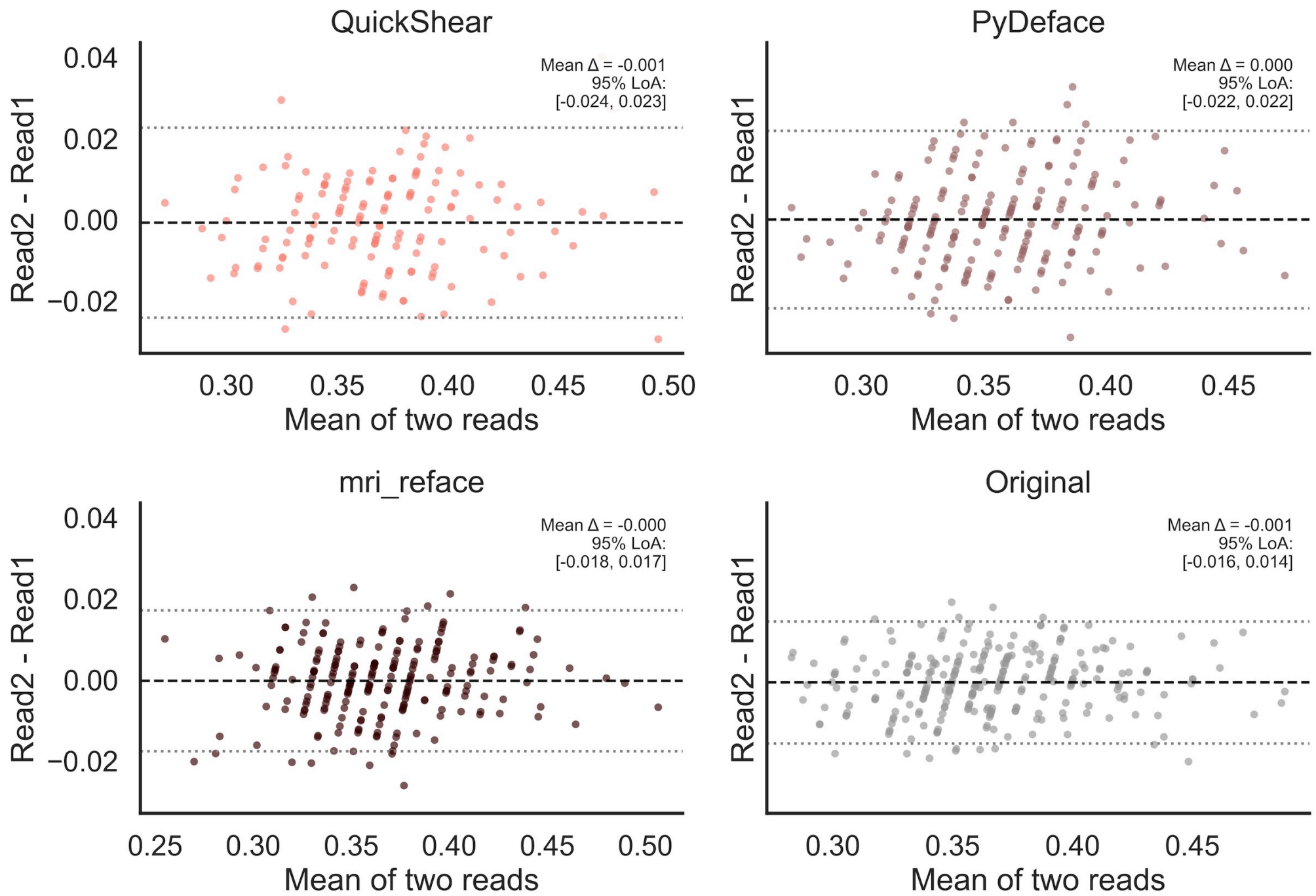

Figure A.10: Intra-reader repeat annotation: first-versus-second Evans ratio measurements across defacing conditions. The dashed diagonal line indicates perfect agreement. All four groups demonstrated strong concordance between repeated measurements, with high correlation coefficients, supporting the overall stability of manual Evans ratio assessment across repeat reads.

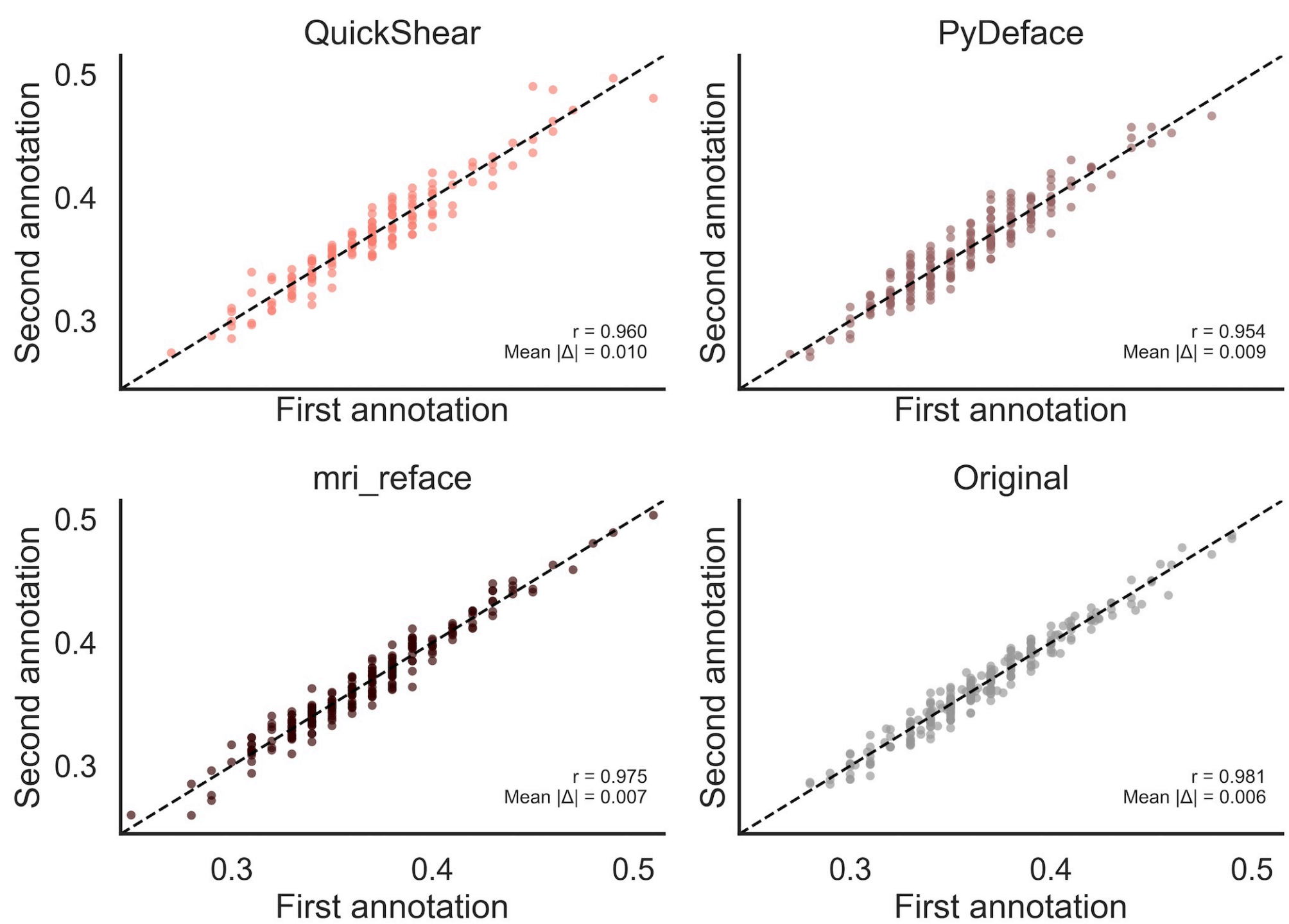

Figure A.11: Bland-Altman analysis of repeat Evans ratio annotation across defacing conditions.
